# The m6A Reader IGF2BP2 Drives Depressive-like Behaviors Through m6A-Dependent Repression of Astrocytic GLUT1 and Glucose Metabolism

**DOI:** 10.3390/ijms27219803

**Published:** 2026-10-31

**Authors:** Zijing Liu, Jiayu Li, Kexin Meng, Yuwen Su, Yaqun Zhang, Jianbo Xiu

**Affiliations:** 1 Institute of Basic Medical Sciences, Chinese Academy of Medical Sciences, School of Basic Medicine Peking Union Medical College, Beijing 100005, China; cynthia_lzj@163.com (Z.L.);; 2 State Key Laboratory of Common Mechanism Research for Major Diseases, Beijing 100005, China; 3 Department of Urology, Peking University First Hospital, Beijing 100034, China; 4 Beijing Key Laboratory of Urogenital Diseases (Male) Molecular Diagnosis and Treatment Center, Beijing 100034, China; 5 National Urological Cancer Center, Beijing 100034, China; 6 State Key Laboratory of Complex, Severe, and Rare Diseases, Beijing 100005, China

**Keywords:** RNA modification, N6-methyladenosine, epigenetic regulation, major depressive disorder, chronic unpredictable mild stress, glucose metabolism

## Abstract

Major depressive disorder (MDD) is a severe psychiatric condition with complex etiology and limited therapeutic targets. In this study, we identify the m6A reader protein IGF2BP2 as a critical epigenetic regulator in depression. We demonstrate that IGF2BP2 expression is significantly upregulated in the hippocampus of both MDD patients and mice subjected to chronic unpredictable mild stress (CUMS). Further, hippocampal knockdown of *Igf2bp2* ameliorated depressive-like behaviors in mice. Mechanistically, we demonstrated that the IGF2BP2 protein, specifically in astrocytes, binds to the mRNA of *glucose transporter 1* (*GLUT1*) and regulates the expression of GLUT1 in an m6A-dependent manner, mainly by promoting its decay, leading to impaired astrocytic glucose uptake. Moreover, overexpression of *Glut1* rescued both glucose metabolic deficits and depressive-like behaviors. Taken together, our findings reveal a previously unrecognized IGF2BP2-GLUT1 axis in the pathogenesis of depression and highlight its potential as a therapeutic target for antidepressant intervention.

## 1. Introduction

Major depressive disorder (MDD) is currently one of the leading causes of disability and death worldwide [1]. It is characterized by persistent low mood, diminished interest in daily activities, cognitive dysfunction, and suicidal behaviors [2]. Both genetic and environmental factors play roles in the pathogenesis of MDD [3]. Despite decades of research, the mechanism of MDD pathogenesis and progression remains incompletely resolved, largely due to heterogeneity in etiology and lack of objective biomarkers. Recently, numerous studies have shown that epigenetic modifications may be potential biomarkers and play a crucial role in the development of MDD, including DNA methylation [4,5], histone modifications [6,7,8], and miRNA [9,10,11]. However, the regulatory role of RNA methylation on the pathogenesis and progression of MDD remains largely elusive.

N6-methyladenosine (m6A) methylation is the most abundant internal modification of eukaryotic mRNA and plays critical roles in multiple fundamental biological processes [12,13]. The dynamic and reversible nature of m6A modification in mammals is orchestrated by the methyltransferase complex (“m6A writer”) and demethylase proteins (“m6A erasers”). The canonical writer complex consists of the METTL3/METTL14/WTAP core along with various co-factors [14,15,16], while erasers include FTO and ALKBH5 [17,18]. The downstream functions of m6A are executed by effector proteins, commonly designated as “m6A readers”. Proteins harboring the YT521-B homology (YTH) domain constitute a well-characterized reader family that governs pre-mRNA splicing, mRNA decay, and mRNA translation [19,20,21,22,23]. A distinct class of readers was uncovered subsequently: the insulin-like growth factor-2 (IGF2) mRNA-binding proteins 1, 2, and 3 (IGF2BP1/2/3) [24]. These IGF2BPs are organized with six canonical RNA-binding domains, specifically two RNA recognition motif (RRM) domains and four K homology (KH) domains [25,26]. Although the regulatory functions of the YTH domain-containing m6A reader proteins have been well characterized in diverse biological contexts and disease processes, such as cell differentiation, tissue development, and tumorigenesis [19,20,21,22,23], the roles of the IGF2BPs remain less extensively explored.

IGF2BP2 is a multifunctional RNA-binding protein that acts as a reader of m6A modification on target mRNAs. It typically binds to m6A-modified transcripts via its C-terminal KH domains, particularly the KH3-4 domains, thereby regulating mRNA stability and translation [27]. IGF2BP2 is enriched in dendrites and plays a pivotal role in processes ranging from neural development and synaptic plasticity to learning and memory formation [28]. For instance, it is hypothesized to stabilize transcripts encoding essential synaptic proteins, thereby facilitating long-term memory consolidation [29]. Besides, compelling evidence also demonstrated that IGF2BP2 in the brain could regulate neuronal differentiation, neuroinflammation, neurotoxicity and the pathogenesis of various neurological disorders [30]. Its function as an N6-methyladenosine (m6A) reader has also been associated with neurodevelopmental disorders and Alzheimer’s disease [30,31]. However, the mechanism by which IGF2BP2 might regulate the pathogenesis and progression of depression has not yet been elucidated.

Beyond the biological characterization of m6A machinery components, accumulating evidence has shown that epitranscriptomic modifications are highly correlated with mood disorders. A recent systematic review highlighted several specific links between m6A methylation and MDD, including stress-induced remodeling of m6A in the prefrontal cortex and amygdala, glucocorticoid-driven dysregulation of m6A in peripheral blood of MDD patients, and genetic associations between MDD and polymorphisms in the m6A demethylase genes *FTO* and *ALKBH5* [32]. Consistent with this framework, candidate gene analysis identified the association between rs9939609 polymorphism in the m6A demethylase gene *FTO* and MDD risk, particularly the atypical subtype [33,34]. A subsequent study of 23 SNPs across five m6A-related genes (*ALKBH5*, *METTL3*, *METTL14*, *WTAP*, *FTO*) in a Chinese Han cohort further demonstrated that *ALKBH5* variants were significantly associated with MDD, and additional SNPs in m6A machinery genes correlated with specific symptom dimensions such as anxiety, retardation, and cognitive disturbance [35].

More direct molecular evidence for m6A dysregulation in depression came from transcriptomic and functional analyses. Researchers reported that acute restraint stress induces brain region-specific remodeling of the m6A epitranscriptome in mice, with decreased methylation in the prefrontal cortex and increased methylation in the amygdala; moreover, m6A levels were dysregulated in peripheral blood of MDD patients following glucocorticoid stimulation [36]. Besides, a profiling study of the dorsolateral prefrontal cortex in MDD subjects identified over 1200 hypermethylated and nearly 7000 hypomethylated transcripts, accompanied by reduced FTO and increased METTL3 expression, and further revealed distinct m6A signatures associated with suicide [37]. Moreover, sexually dimorphic m6A patterns have also been observed. One of the recent studies characterized transcriptome-wide m6A alterations in the ventromedial prefrontal cortex of MDD patients and found that dysregulated methylation was linked to microtubule movement pathways in males and neuronal projection pathways in females [38].

Functional studies have provided further causal evidence linking specific m6A regulators to depressive-like behaviors. For example, Huang et al. reported that circular RNA circSTAG1 attenuates depressive-like behaviors by blocking the nuclear translocation of the m6A demethylase ALKBH5 in astrocytes, thereby elevating m6A methylation of *FAAH* mRNA and promoting its degradation [39]. Recent reviews have further synthesized the growing evidence linking m6A machinery to psychiatric conditions, including depression, anxiety, schizophrenia, and bipolar disorder, and highlighted the therapeutic potential of targeting the m6A machinery components [40,41].

Despite this progress, the contribution of specific m6A reader proteins—especially the IGF2BP family—to mood disorder pathogenesis has remained largely unexplored. Given the emerging link between cerebral glucose hypometabolism and the pathophysiology of MDD [42], elucidating the role of metabolic dysregulation in epigenetic mechanisms of depression is of particular interest.

Here, we report that IGF2BP2 in the hippocampus mediates depressive-like behaviors through m6A modification reader protein activity. The expression of hippocampal IGF2BP2 is increased in MDD patients and CUMS mice compared with controls. Knockdown of *Igf2bp2* in the mice hippocampus induces antidepressant behaviors. Further study identified *GLUT1* as a target mRNA of IGF2BP2, with IGF2BP2 mediating its regulation in an m6A-dependent manner. GLUT1 activation rescues depressive-like behaviors and glucose uptake deficiency caused by elevated hippocampal IGF2BP2 expression. It is concluded that hippocampal IGF2BP2 may be a potential therapeutic target for depression.

## 2. Results

### 2.1. The Expression of m6A Reader IGF2BP2 Is Upregulated in the Peripheral Blood and Hippocampus of MDD Patients and CUMS Mice

**Figure 1:**
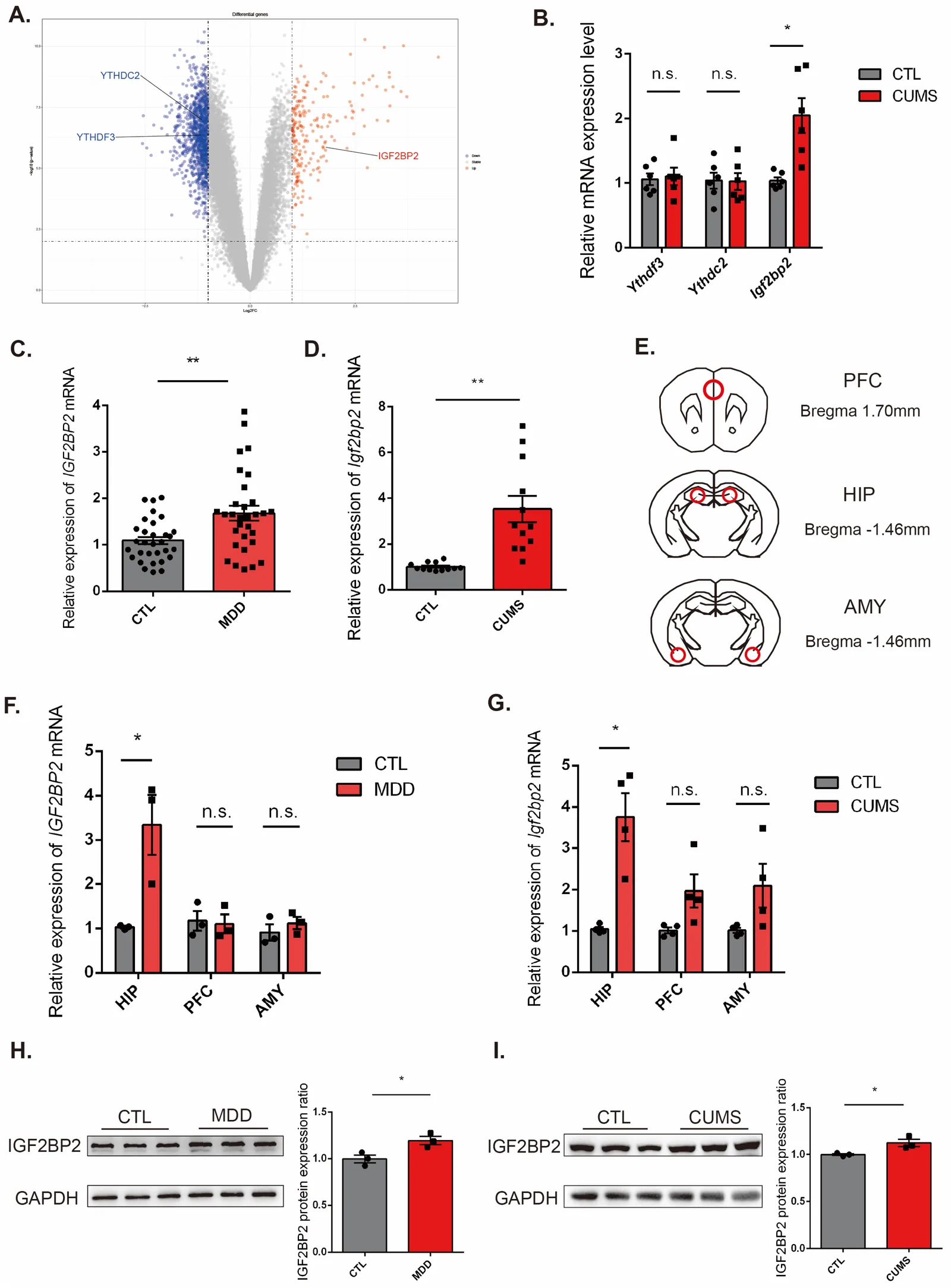
Expression of m6A reader IGF2BP2 is increased in the peripheral blood and hippocampus of major depressive disorder (MDD) patients and chronic unpredictable mild stress (CUMS) mice. (**A**) Volcano plot for differential expression analysis of microarrays (peripheral blood of 20 MDD patients and 20 healthy controls). Vertical and horizontal dashed lines represent the thresholds for discovering differentially expressed m6A RNA modification regulatory genes (absolute Log2 fold change >1 and *p* value < 0.01). Red dots indicate upregulated genes, blue dots indicate downregulated genes (MDD patients compared with healthy controls); (**B**) qRT-PCR assay for *Ythdf3*, *Ythdc2* and *Igf2bp2* expression in the peripheral blood of CUMS and control mice. CTL, *n* = 6; CUMS, *n* = 6; (**C**) Verification of the elevated *IGF2BP2* mRNA expression by RT-qPCR in an independent cohort (peripheral blood of 31 MDD patients and 31 healthy controls). (**D**) Verification of the elevated *IGF2BP2* mRNA expression in the peripheral blood of CUMS mice. CTL, *n* = 12; CUMS, *n* = 12; (**E**) Schematic diagram shows different brain regions of mice. PFC, prefrontal cortex; HIP, hippocampus; AMY, amygdaloid nucleus; (**F**) qRT-PCR assay for *IGF2BP2* mRNA expression in different brain regions of MDD patients’ postmortem brains. CTL, *n* = 3; MDD, *n* = 3; (**G**) qRT-PCR assay for *Igf2bp2* mRNA expression in different brain regions of CUMS mice. CTL, *n* = 4; CUMS, *n* = 4; (**H**) Western blot assay of IGF2BP2 protein expression in the hippocampus of MDD patients. CTL, *n* = 3; MDD, *n* = 3; (**I**) Western blot assay of IGF2BP2 protein expression in the hippocampus of CUMS mice. CTL, *n* = 3; CUMS, *n* = 3; (**C**–**I**): Grey bars represent the control (CTL) group, and red bars represent the MDD (or CUMS) group. Individual data points are shown as circles (CTL) and squares (MDD/CUMS), representing individual biological replicates. * *p* < 0.05, ** *p* < 0.01, n.s. not significant. Error bars represent the standard error of the mean (s.e.m.).

We conducted transcriptome microarray profiling of peripheral blood samples from 20 patients with major depressive disorder (MDD) and 20 healthy individuals to explore the potential involvement of m6A readers in MDD pathogenesis (detailed information about the MDD patients and healthy controls is listed in the Supplementary Table). We found that three m6A readers were differentially expressed in the patients group, with two downregulated and one upregulated (Figure 1A). Next, we examined the expression of these m6A readers in CUMS model mice in order to investigate whether these expression variations in MDD patients can be replicated in the mice model of depression. In the peripheral blood of CUMS mice, *Igf2bp2* mRNA was significantly upregulated relative to control mice, whereas *Ythdf3* and *Ythdc2* mRNA expression remained unchanged (Figure 1B).

To further validate the upregulation of *IGF2BP2*, we next examined the mRNA expression of *IGF2BP2* by RT-qPCR in another independent cohort with 31 MDD patients and 31 healthy controls (detailed information is listed in the Supplementary Table), and a new CUMS model with 12 CUMS mice and 12 control mice. Behavioral tests, including the forced swimming test (FST) and sucrose preference test (SPT), showed that the two CUMS mice models of depression were successfully established (Figure S1A,B). We found that *IGF2BP2* mRNA expression was also upregulated in the peripheral blood of both MDD patients and CUMS mice (Figure 1C,D).

We subsequently examined *IGF2BP2* mRNA expression in principal brain regions implicated in emotional regulation, with a focus on the prefrontal cortex (PFC), hippocampus (HIP) and amygdala (AMY) (Figure 1E). We found that the *IGF2BP2* mRNA expression was significantly upregulated in both the postmortem hippocampus of MDD patients and CUMS mice, whereas no significant changes were noted in other brain regions (Figure 1F,G). Western blot analysis further confirmed that IGF2BP2 was significantly upregulated in the hippocampus of MDD patients (Figure 1H) and CUMS mice (Figure 1I), compared with healthy controls and control mice, respectively.

### 2.2. Hippocampal Knockdown of Igf2bp2 Ameliorates Depressive-like Behaviors in CUMS Mice

**Figure 2:**
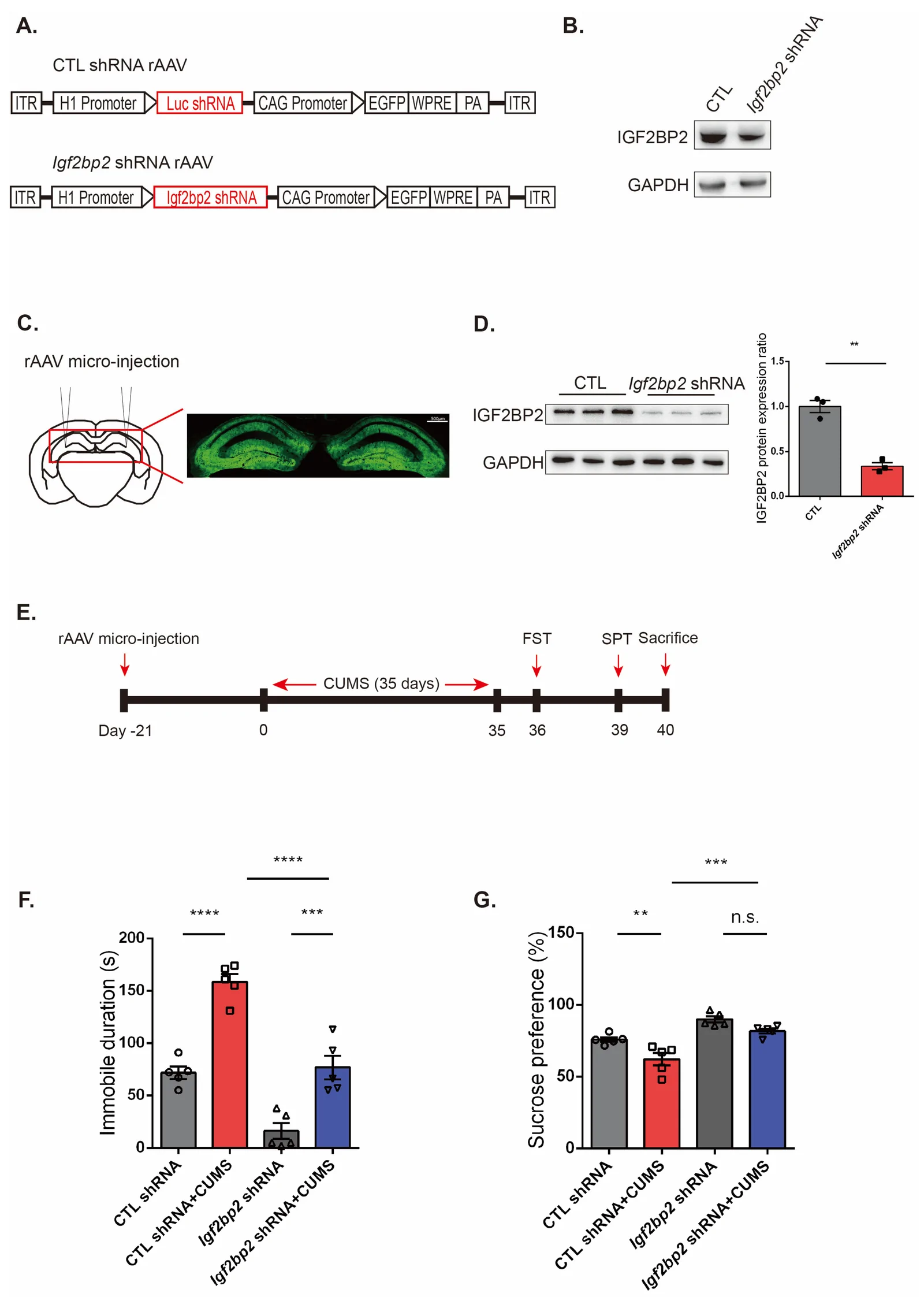
*Igf2bp2* knockdown rescues depressive-like behaviors of CUMS mice. (**A**) Schematic diagram of the rAAV vectors expressing control (Ctrl) shRNA and *Igf2bp2* shRNA. (**B**) Validation of the *Igf2bp2* knockdown efficiency in N2A cells. (**C**) Validation of *Igf2bp2* shRNA virus infection into the hippocampus indicated by EGFP fluorescence. (Scale bar, 500 μm) (**D**) Western blot assay of IGF2BP2 expression in the hippocampus of mice after *Igf2bp2* shRNA virus microinjection into hippocampus. (**E**) Schematic schedule diagram of the CUMS modeling and behavior tests. (**F**) Forced swimming test. *n* = 5 per group. (**G**) Sucrose preference test. *n* = 5 per group. (**F**–**G**): The grey bars represent the shRNA conditions (circles for CTL shRNA; upright triangles for *Igf2bp2* shRNA), while the red and blue bars represent the CTL shRNA + CUMS and *Igf2bp2* shRNA + CUMS groups (squares and inverted triangles, respectively). Symbols represent individual biological replicates. ** *p* < 0.01, *** *p* < 0.001, **** *p* < 0.0001, n.s. not significant. Error bars represent s.e.m.

To determine whether elevated hippocampal *Igf2bp2* contributes to the development of depressive-like behaviors, we utilized the recombinant adeno-associated virus (rAAV) mediated gene transfer approach to specifically knock down *Igf2bp2* in the hippocampus of mice (Figure 2A) We first verified the efficiency of *Igf2bp2* shRNA in N2A cells (Figure 2B). Specifically, 21 days after bilateral hippocampal rAAV microinjection, the *Igf2bp2* shRNA-expressing mice displayed a significant reduction in hippocampal IGF2BP2 protein level compared to Ctrl shRNA-expressing mice, indicating the high efficiency of *Igf2bp2* knockdown after rAAV microinjection (Figure 2D). The efficiency of rAAV infection was also validated by EGFP fluorescence (Figure 2C).

To evaluate the antidepressant potential of downregulating the elevated *Igf2bp2* levels in the hippocampus of depressive-like mice, we initiated the CUMS paradigm 21 days after intrahippocampal delivery of rAAV. After 35 days of the CUMS procedure, we analyzed the behavioral consequences of *Igf2bp2* expression changes (Figure 2E). Relative to unstressed Ctrl shRNA-expressing mice, mice that received Ctrl shRNA and underwent the CUMS paradigm exhibited a significant prolongation of immobility time in the forced swimming test (FST) and a marked reduction in sucrose preference in the sucrose preference test (SPT), indicating depressive-like behaviors induced by CUMS (Figure 2F,G). In addition, compared with the control group, mice with hippocampal *Igf2bp2* knockdown showed significantly shorter immobility time in FST and elevated sucrose preference in SPT, supporting the conclusion that downregulation of *Igf2bp2* in the mice hippocampus exerted antidepressant effects (Figure 2F,G).

### 2.3. GLUT1 Is a Target Gene of IGF2BP2 in the Hippocampus in Depression

**Figure 3:**
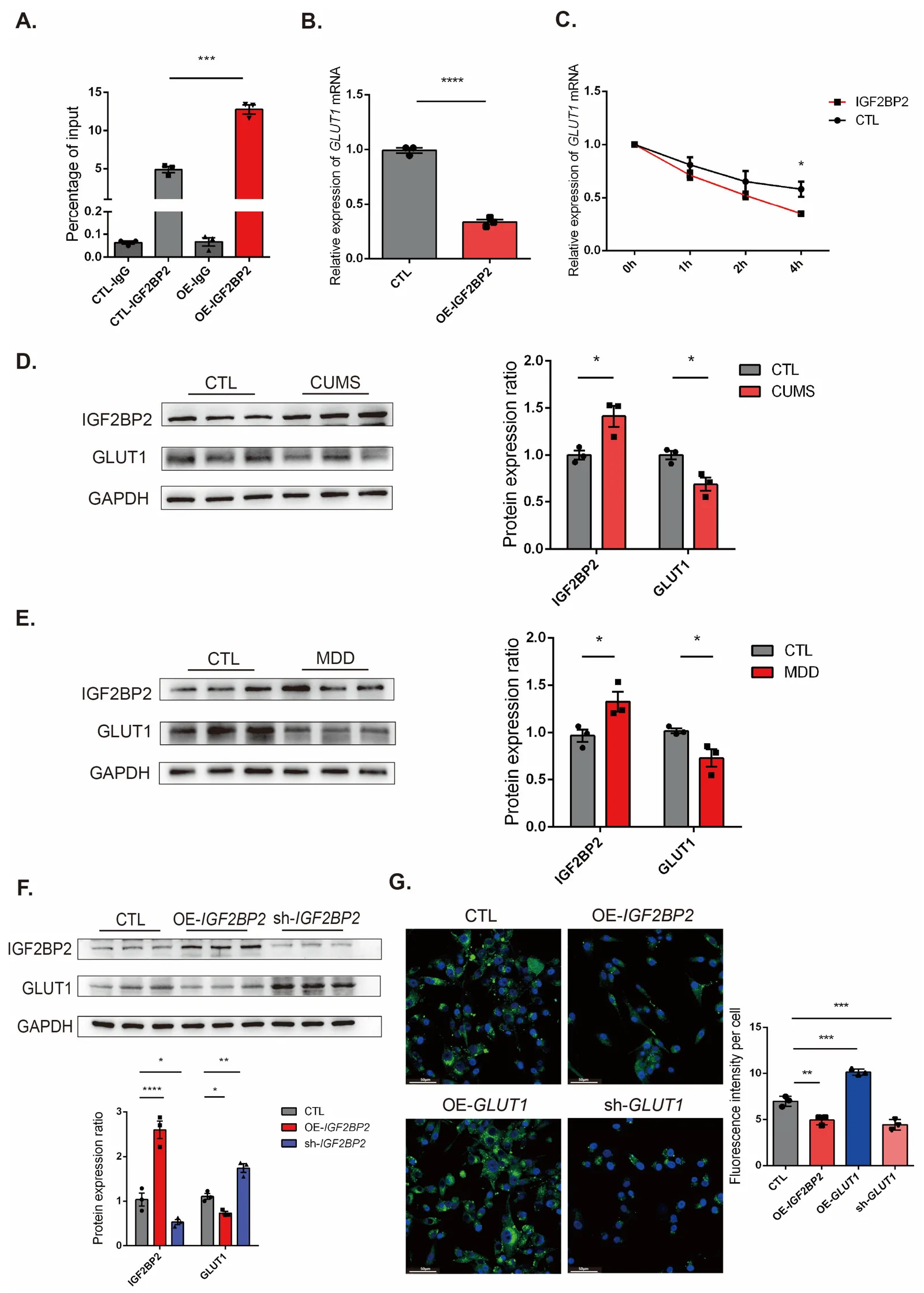
*Glucose transporter 1* (*GLUT1*) is one of the critical target mRNAs of IGF2BP2 in the hippocampus in depression. (**A**) The IGF2BP2 binding level of *GLUT1* mRNA in U87 cells detected by RIP-qPCR. CTL-IgG antibody group is shown with grey bars and circle symbols; CTL-IGF2BP2 antibody group with grey bars and square symbols; OE-IgG antibody group with grey bars and upright triangle symbols; OE-IGF2BP2 antibody group with red bars and inverted triangle symbols. Individual symbols represent biological replicates. (**B**) qPCR assay of *GLUT1* mRNA expression in U87 cells overexpressing IGF2BP2. Grey bars represent the control (CTL) group; red bars represent the overexpression group (OE-IGF2BP2). Individual data points are shown as circles (CTL) and squares (OE-IGF2BP2), representing individual biological replicates. (**C**) *GLUT1* mRNA stability assay of U87 cells overexpressing *IGF2BP2*. (**D**) Western blot assay of GLUT1 protein expression in the hippocampus of CUMS mice. (**E**) Western blot assay of IGF2BP2 protein expression in the hippocampus of MDD patients. (**F**) Western blot assay of IGF2BP2 and GLUT1 protein expression in U87 cells with *IGF2BP2* overexpression or knockdown. (**G**) 2-NBDG assay of glucose uptake in U87 cells with altered IGF2BP2 or GLUT1 expression. Green fluorescence indicates the 2-NBDG signal, representing glucose uptake, while blue fluorescence indicates DAPI nuclear counterstain. (Scale bar, 50 μm). In the quantitative bar graph, CTL group is shown with grey bars and circle symbols; OE-IGF2BP2 group with red bars and square symbols; the OE-GLUT1 group with blue bars and upright triangle symbols; sh-GLUT1 group with pink bars and inverted triangle symbols. Individual symbols represent biological replicates. * *p* < 0.05, ** *p* < 0.01, *** *p* < 0.001, **** *p* < 0.0001. Error bars represent s.e.m.

In order to shed light on the role of elevated IGF2BP2, we further tried to identify a functional target mRNA of IGF2BP2 in depression, as IGF2BP2 is an mRNA-binding protein. Several studies have shown that IGF2BP2 recognizes and binds to the m6A site of the insulin-independent *glucose transporter 1* (*GLUT1*) mRNA and regulates its stability in different types of cancer cells [43,44]. GLUT1 plays a pivotal role in brain metabolism and regulating the glucose uptake of astrocytes, which is a major type of cell in hippocampus. We then examined whether *GLUT1* is one of the critical target genes of IGF2BP2 in depression. RNA immunoprecipitation (RIP) qPCR assays showed the effective binding of overexpressed IGF2BP2 to *GLUT1* mRNA in U87 cells, which are adherent astrocytes derived from human malignant glioma. (Figure 3A).

Therefore, we further examined whether GLUT1 in the hippocampus contributed to IGF2BP2-mediated depressive-like behaviors. First, we confirmed that IGF2BP2 overexpression led to decreased *GLUT1* mRNA expression in U87 cells (Figure 3B). In the presence of actinomycin D, an inhibitor of mRNA transcription, IGF2BP2 overexpression significantly accelerated the degradation of *GLUT1* mRNA (Figure 3C). These results indicate that IGF2BP2 may affect *GLUT1* mRNA expression by regulating its stability.

Next, we tested the GLUT1 protein levels in the hippocampus of both CUMS mice and MDD patients. We found that protein expression of GLUT1 was significantly lower in the hippocampus of CUMS mice than control mice, while IGF2BP2 was significantly elevated (Figure 3D). The postmortem study also showed decreased GLUT1 expression in the hippocampus of MDD patients compared to healthy controls, while IGF2BP2 was significantly elevated (Figure 3E). In order to confirm the regulating role of IGF2BP2 on GLUT1, we further conducted *IGF2BP2* overexpression and *IGF2BP2* knockdown in U87 cells. We found that the GLUT1 protein was significantly decreased in U87 cells with *IGF2BP2* overexpression and increased in U87 cells with *IGF2BP2* knockdown (Figure 3F).

Given that GLUT1 plays a critical role in regulating the glucose uptake of astrocytes, we hypothesized that the elevated IGF2BP2 in the hippocampus during depression suppresses astrocytic glucose uptake through the downregulation of GLUT1. To examine this hypothesis, we utilized the fluorescent glucose analog 2-NBDG, which is taken up by cells via the same transport machinery as glucose. Since 2-NBDG is not a substrate for glycolytic metabolism, it is retained within the cytoplasm, making it a useful indicator of glucose uptake activity. This accumulation allows visualization through confocal fluorescence microscopy. The results showed that U87 cells with *IGF2BP2* overexpression exhibited decreased glucose uptake, consistent with U87 cells with *GLUT1* knockdown, while U87 cells with *GLUT1* overexpression showed increased glucose uptake compared with control (Figure 3G).

Taken together, these results indicated that *GLUT1* may be a target mRNA of IGF2BP2 in depression. The elevated IGF2BP2 expression in the hippocampus during depression may lead to decreased GLUT1 expression, finally impairing the uptake of glucose in the astrocytes.

### 2.4. IGF2BP2 Regulates GLUT1 Expression in an m6A-Dependent Manner

**Figure 4:**
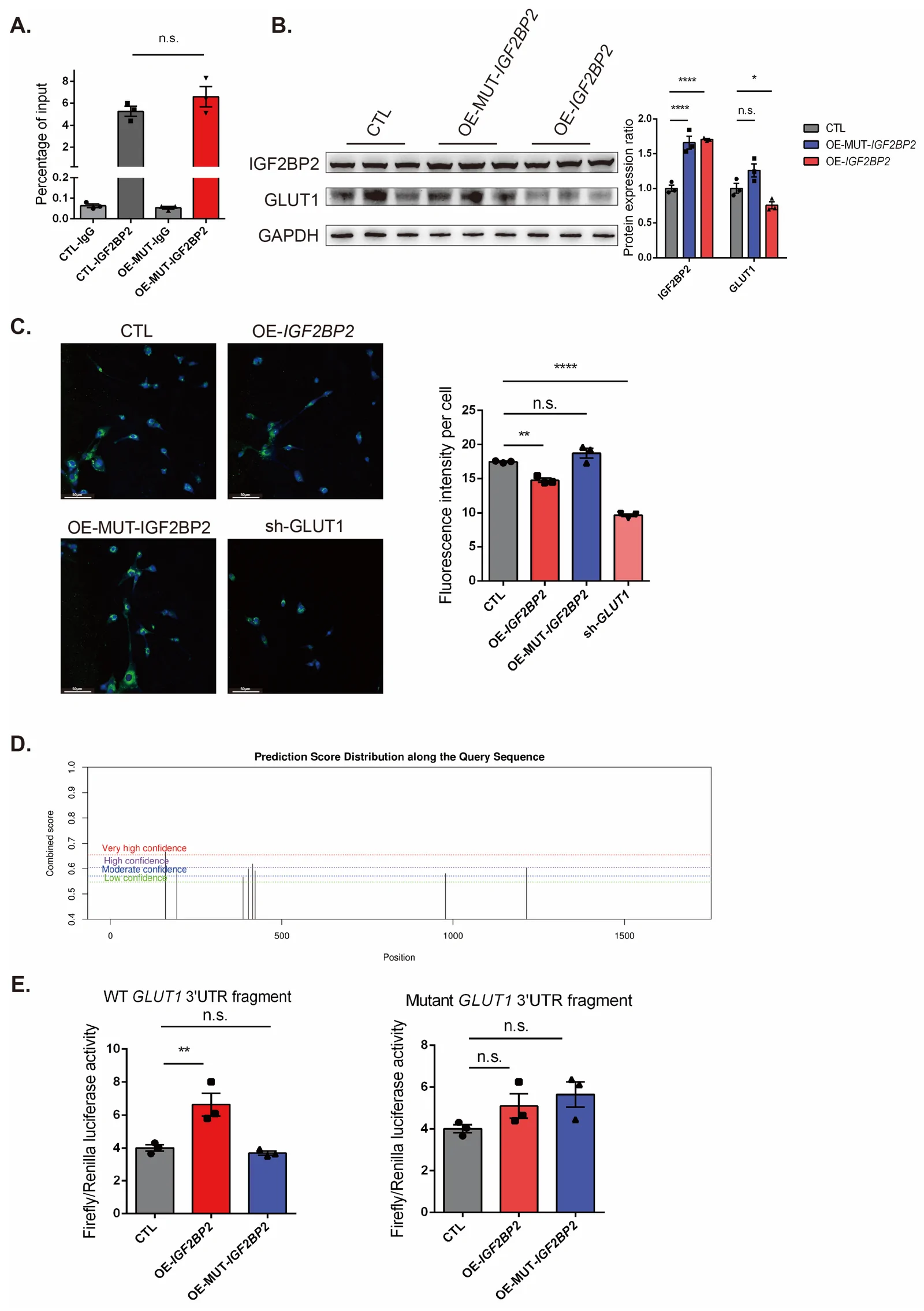
IGF2BP2 regulates GLUT1 expression in an m6A-dependent manner. (**A**) The KH3-4 domain mutant IGF2BP2 binding level of *GLUT1* mRNA in U87 cells detected by RIP-qPCR. CTL-IgG antibody group is shown with grey bars and circle symbols; CTL-IGF2BP2 antibody group with grey bars and square symbols; OE-mutant IGF2BP2-IgG antibody group with grey bars and upright triangle symbols; OE-mutant IGF2BP2-IGF2BP2 antibody group with red bars and inverted triangle symbols. Individual symbols represent biological replicates. (**B**) Western blot assay of IGF2BP2 and GLUT1 protein expression in U87 cells with IGF2BP2 overexpression or KH3-4 domain mutant IGF2BP2 overexpression. (**C**) 2-NBDG assay of glucose uptake in U87 cells with IGF2BP2 overexpression or KH3-4 domain mutant IGF2BP2 overexpression. Green fluorescence indicates the 2-NBDG signal, representing glucose uptake, while blue fluorescence indicates DAPI nuclear counterstain. (Scale bar, 50 μm) In the quantitative bar graph, CTL group is shown with grey bars and circle symbols; OE-IGF2BP2 group with red bars and square symbols; OE-MUT-IGF2BP2 group with blue bars and upright triangle symbols; sh-GLUT1 group with pink bars and inverted triangle symbols. Individual symbols represent biological replicates. (**D**) The m6A methylation site of *GLUT1* mRNA was predicted using the SRAMP website. (**E**) Relative luciferase activity of pMIR-GLO-GLUT1-3′UTR with either wild-type (WT *GLUT1* 3′UTR fragment) or mutant m6A sites (Mutant *GLUT1* 3′UTR fragment) after co-transfection with control vector (CTL), wildtype IGF2BP2 (OE-IGF2BP2), or KH3-4 domain mutant IGF2BP2 (OE-MUT-IGF2BP2) for 48 h in U87 cells. Firefly luciferase activity was measured and normalized to Renilla luciferase activity, *n* = 3 per group. CTL group is shown with grey bars and circle symbols; OE-IGF2BP2 group with red bars and square symbols; OE-mutant-IGF2BP2 group with blue bars and upright triangle symbols. Individual symbols represent biological replicates. * *p* < 0.05, ** *p* < 0.01, **** *p* < 0.0001, n.s. not significant. Error bars represent s.e.m.

Given the fact that IGF2BP2 is a typical m6A reader protein and our previous results indicated the direct binding and regulation of IGF2BP2 on *GLUT1* mRNA, we further verified whether IGF2BP2 regulated *GLUT1* in an m6A-dependent manner. First, we generated an IGF2BP2 mutant harboring mutations in the m6A recognition sites by converting GxxG to GEEG in both KH3 and KH4 domain of IGF2BP2, which are indispensable for m6A recognition. RIP qPCR assays showed that the effective binding of the IGF2BP2 protein to *GLUT1* mRNA in U87 cells was largely abolished after mutating the m6A-recognition domain of IGF2BP2 (Figure 4A). Western blot assay further indicated that the m6A-recognition domain mutant IGF2BP2 no longer regulated GLUT1 expression (Figure 4B). In addition, we performed 2-NBDG glucose uptake assays and found that the mutant IGF2BP2 overexpression failed to decrease the glucose uptake of U87 cells compared with wild type IGF2BP2 overexpression (Figure 4C).

Next, we predicted the potential m6A modification sites on *GLUT1* mRNA by SRAMP, an online m6A site predictor, and converted “GGACU” to “GGCCU” on the *GLUT1* mRNA, which is the predicted m6A modification site with very high confidence (Figure 4D). To assess whether the binding of IGF2BP2 to *GLUT1* mRNA occurs in an m6A-dependent manner, we employed luciferase reporter assays. Compared with either the empty vector or a mutant IGF2BP2, wild-type IGF2BP2 significantly enhanced the luciferase signal of the pMIR-GLO constructs carrying *GLUT1* 3′UTR with wild-type m6A sites. In contrast, wild-type IGF2BP2 did not influence the activity of the pMIR-GLO vectors bearing the *GLUT1* 3′UTR with the mutant m6A site (Figure 4E). Such data indicated that IGF2BP2 protein bound to the *GLUT1* mRNA and regulated the expression and function of *GLUT1* in an m6A modification-dependent way.

### 2.5. Hippocampal Overexpression of Glut1 Ameliorates Depressive-like Behaviors in CUMS Mice

**Figure 5:**
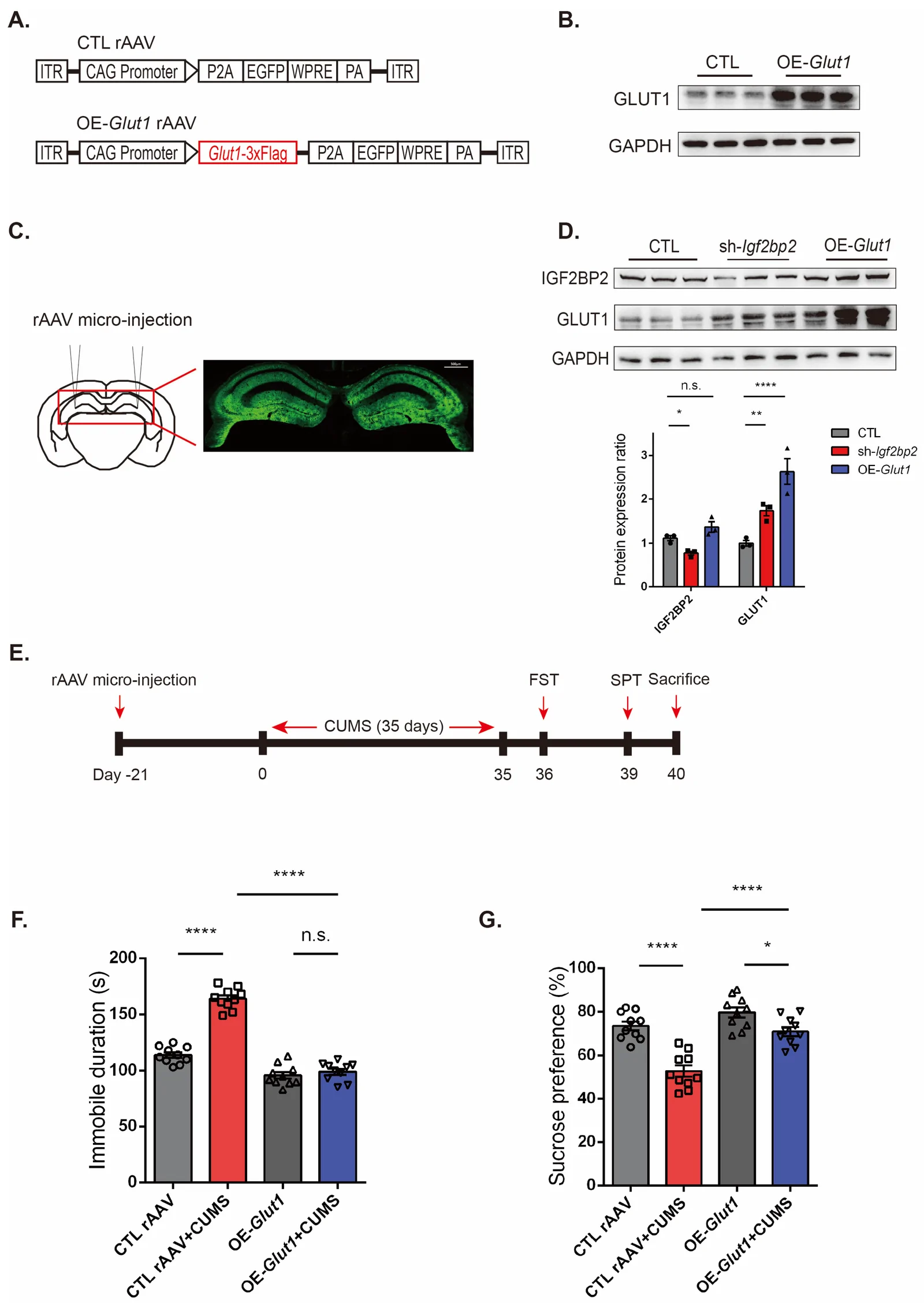
*Glut1* overexpression rescues depressive-like behaviors induced by *Igf2bp2* upregulation in the hippocampus of mice. (**A**) Schematic diagram of the rAAV vectors expressing control (Ctrl) and *Glut1* overexpression (OE-Glut1) vector. (**B**) Western blot assay of hippocampal GLUT1 expression of mice after *Glut1* overexpression virus microinjection. (**C**) Validation of *Glut1* overexpression virus infection into the hippocampus indicated by EGFP fluorescence. (Scale bar, 500 μm) (**D**) Western blot assay of IGF2BP2 and GLUT1 protein expression in mice hippocampus with *Igf2bp2* knockdown or *Glut1* overexpression. (**E**) Schematic schedule diagram of the CUMS modeling and behavior tests. (**F**) Forced swimming test. *n* = 10 per group. (**G**) Sucrose preference test. *n* = 10 per group. (**F**–**G**): The grey bars represent groups without CUMS conditions (circles for CTL; upright triangles for OE-*Glut1*), while the red and blue bars represent the CTL with CUMS and OE-*Glut1* with CUMS groups (squares and inverted triangles, respectively). Symbols represent individual biological replicates. * *p* < 0.05, ** *p* < 0.01, **** *p* < 0.0001, n.s. not significant. Error bars represent s.e.m.

To test whether decreased expression of GLUT1 in the hippocampus, which is induced by elevated IGF2BP2 expression, contributes to depressive-like behaviors, we employed rAAV again as a gene transfer vector to specifically overexpress *Glut1* in the hippocampus of mice (Figure 5A). Specifically, 21 days after microinjection of rAAV into the bilateral hippocampus, the protein level of GLUT1 was significantly increased in the hippocampus of *Glut1*-overexpressing mice compared to control, indicating the high efficiency of *Glut1* overexpression after rAAV microinjection (Figure 5B). The efficiency of rAAV infection was also validated by EGFP fluorescence (Figure 5C). In addition, the Western blot assays showed that the protein level of GLUT1 was significantly increased in the hippocampus of mice after *Igf2bp2* knockdown, while the protein level of IGF2BP2 was unchanged in the hippocampus of mice after *Glut1* overexpression (Figure 5D). These data indicated that IGF2BP2 regulates the expression of its downstream target *Glut1*, but *Glut1* does not reciprocally regulate the expression of *Igf2bp2*.

Next, in order to verify the direct regulatory role of GLUT1 on depression, we further treated mice with the CUMS procedure 21 days after rAAV microinjection to test whether upregulating the decreased expression of *Glut1* in the hippocampus of depressive-like mice exerted antidepressant effects. After 35 days of CUMS procedure, we analyzed the behavioral consequences of *Glut1* expression changes (Figure 5E). Mice with hippocampal *Glut1* overexpression demonstrated significant shorter immobility duration in FST and higher sucrose preference in SPT compared with CUMS-treated mice, indicating an antidepressant effect induced by upregulating the expression of *Glut1* in the mice hippocampus (Figure 5F,G).

## 3. Discussion

MDD is a debilitating psychiatric condition with complex and heterogeneous etiology, necessitating the identification of novel molecular mechanisms and therapeutic targets [45,46]. In this study, we provide evidence that the m6A reader protein IGF2BP2 is significantly upregulated in the hippocampus of both MDD patients and CUMS-induced depressive-like mice. Further experiments revealed that hippocampal knockdown of *Igf2bp2* ameliorated depressive-like behaviors of mice, suggesting a causal role of IGF2BP2 in the pathogenesis of depression. Furthermore, we identified *GLUT1*, a key glucose transporter enriched in the brain astrocytes, as a direct downstream target mRNA of IGF2BP2. The expression of GLUT1 was suppressed by IGF2BP2 in an m6A reader-dependent manner. Importantly, overexpression of *Glut1* in the hippocampus rescued both behavioral deficits and glucose uptake impairment, highlighting the functional significance of the IGF2BP2-GLUT1 axis in depression.

Our findings identified IGF2BP2 as a critical epigenetic regulator in depression. Although previous studies have implicated m6A modifications in various neuropsychiatric disorders [47], the specific role of IGF2BP2 in mood disorders remained unexplored. We found that IGF2BP2 expression is elevated specifically in the hippocampus, a brain region central to emotional regulation and cognitive function, in both human postmortem tissue and a validated CUMS mouse model of depression. The behavioral rescue achieved by hippocampal *Igf2bp2* knockdown underscores the functional relevance of this m6A reader in depressive-like phenotypes and highlights the therapeutic potential of the IGF2BP2-GLUT1 axis. However, given that IGF2BP2 is a multifunctional RNA-binding protein that may regulate a broad repertoire of target transcripts beyond *GLUT1*, direct pharmacological inhibition of IGF2BP2 may carry a risk of unintended off-target effects. A more precise intervention, such as selectively enhancing GLUT1 expression or restoring its downstream metabolic function in astrocytes, may therefore represent a safer and more targeted therapeutic strategy.

Notably, we mechanistically linked IGF2BP2 to metabolic dysfunction in astrocytes through its regulation of GLUT1. Using RIP-qPCR, luciferase reporter assays, and mutagenesis studies, we demonstrated that IGF2BP2 binds to *GLUT1* mRNA and accelerates its decay in an m6A-dependent manner. This repression led to impaired glucose uptake, as evidenced by decreased 2-NBDG fluorescence in U87 cells overexpressing IGF2BP2. Given that astrocytic glucose metabolism plays a crucial role in maintaining neuronal energy homeostasis and synaptic function [48], our results suggest that IGF2BP2 overexpression in depression may contribute to glucose uptake deficiency in the hippocampus, thereby exacerbating depression [49].

Alterations in brain glucose metabolism have been recognized as a hallmark of MDD and a potential avenue for MDD therapeutic intervention. Clinical neuroimaging studies using ^18^F-FDG PET have consistently revealed glucose hypometabolism in the prefrontal cortex and hippocampus of patients with MDD, indicating that impaired cerebral glucose utilization is directly associated with the pathophysiology of depression [42]. Importantly, recent work has demonstrated that therapeutic strategies targeting brain glucose metabolism can ameliorate depressive-like phenotypes. In a corticosterone-induced rat model of depression, Pan et al. demonstrated that the conventional antidepressant fluoxetine increased astrocytic glucose uptake and glycolysis by restricting the glucocorticoid receptor (GR)–thioredoxin-interacting protein (TXNIP)–GLUT1 signaling pathway. This finding provided direct evidence that modulation of astrocytic glucose transport machinery may contribute to antidepressant effects [50]. Consistently, TXNIP was previously identified as a key driver of astrocytic glucose hypometabolism in depressive states, with its upregulation leading to reduced plasma membrane translocation of GLUT1 and impaired glucose uptake in astrocytes [51]. Moreover, the broader therapeutic relevance of glucose metabolism in depression was further supported by evidence that insulin-sensitizing agents such as pioglitazone and metformin produced antidepressant effects in both preclinical depression models and MDD patients [52]. Our findings extended these observations by delineating the upstream epigenetic mechanism that IGF2BP2 post-transcriptionally repressed *GLUT1* via m6A-dependent mRNA decay, thereby driving the astrocytic metabolic dysfunction.

The rescue of depressive-like behaviors and glucose uptake upon *Glut1* overexpression further supports the functional importance of the IGF2BP2-GLUT1 axis. The identification of the IGF2BP2-GLUT1 axis also carries translational significance in light of the well-documented limitations of currently available antidepressant therapies. Conventional clinical antidepressants, which primarily target monoaminergic neurotransmission, fail to achieve adequate response in approximately 30–50% of patients, and a substantial proportion of individuals eventually meet criteria for treatment-resistant depression (TRD) [53,54]. A recent metabolomic study of patients with TRD identified potentially treatable metabolic abnormalities, including cerebral folate deficiency and tetrahydrobiopterin metabolism disturbances, further underscoring the mechanistic link between metabolic dysregulation and treatment-resistant depression [55]. These findings collectively indicate that novel therapeutic strategies directed at molecular targets beyond classical monoamine systems are urgently needed. The discovery that IGF2BP2 post-transcriptionally represses *GLUT1* in an m6A-dependent manner to drive depressive-like behaviors therefore represents a previously unrecognized mechanism that may lead to new therapeutic intervention for MDD, particularly for patients with metabolic disorder.

Despite these insights, our study has several limitations. Firstly, regarding the cellular basis of our findings, in vitro mechanistic experiments were conducted using human brain glioblastoma U87 cell line. While U87 cells are widely employed as a model for metabolic studies of astrocytes due to the ease of genetic manipulation and well-characterized glucose transport machinery [56], they are derived from a malignant tumor and may not fully recapitulate the characteristics of primary astrocytes in vivo. Thus, future validation in primary astrocytes is needed to further confirm the regulatory role of IGF2BP2-GLUT1 axis on depression.

Additionally, our in vivo experiments used hippocampal lysates, which do not distinguish between different cell types. While GLUT1 is predominantly expressed in astrocytes, and our in vitro data suggest an astrocytic mechanism of the IGF2BP2-GLU1 axis, it remains unclear whether the involvement of the IGF2BP2-GLUT1 axis in depression is astrocyte-specific in vivo. To address this, future studies employing cell-type-specific manipulations, such as astrocyte-specific *Igf2bp2* knockdown using recombinant adeno-associated virus (rAAV) with a GFAP promoter, or conditional knockout mice, are essential to establish the causal role of the astrocytic IGF2BP2-GLUT1 axis in the behavioral and metabolic phenotypes, and the inclusion of locomotor activity controls will further validate the behavioral specificity of these findings.

Furthermore, it is important to note that all animal experiments in this study were conducted exclusively in male C57BL/6J mice. Given the well-documented sex differences in the prevalence of major depressive disorder, with women exhibiting approximately twofold higher rates than men [57,58], and the growing recognition of sex-specific molecular mechanisms underlying depression, the generalizability of our findings to female subjects remains to be established. Notably, sexually dimorphic patterns of m6A methylation have recently been reported in the prefrontal cortex of MDD patients, with distinct m6A profiles between males and females [38]. In addition, emerging evidence indicates that sex is a critical variable in m6A-mediated regulation: FTO depletion was shown to impair object location memory in male but not female mice, directly demonstrating sex-dependent effects of m6A demethylase activity on brain function [59]. Sex hormones such as estrogen have also been implicated in the regulation of astrocytic energy metabolism. Studies have shown that estradiol exerts sex-dimorphic control over hypothalamic astrocyte glycogen metabolism, with female astrocytes exhibiting greater sensitivity to estrogen stimulation than male astrocytes [60]. Taken together, it is therefore plausible that the IGF2BP2-GLUT1 axis may operate differently in female mice, or that compensatory mechanisms involving estrogen signaling may exist. Future studies incorporating both male and female animals are warranted to determine whether the IGF2BP2-GLUT1-mediated mechanism is conserved across sexes, and whether sex-specific therapeutic strategies targeting this pathway should be considered.

Moreover, the sample size for human postmortem analysis was modest, and larger cohorts will be necessary to confirm the robustness of our findings. Additionally, for hippocampal Western blot analysis in the CUMS model, three animals per group were randomly selected from the larger behavioral cohorts. While this sampling approach is common in rodent brain tissue studies and proved sufficient to detect the robust protein-level changes reported here, the small number of replicates may limit the statistical power to detect more subtle molecular alterations. Future studies employing larger sample sizes for biochemical endpoints would further strengthen the generalizability of these findings.

In addition, while the CUMS model is well-validated, it does not capture the full heterogeneity of human depression. Complementary models or patient-derived induced pluripotent stem cells (iPSCs) could provide further evidence.

Looking forward, we seek to identify more mRNA targets of IGF2BP2 in depression, which may reveal broader regulatory networks, in order to explore more diagnostic and prognostic biomarkers for MDD. To this end, future studies should aim to comprehensively profile the full spectrum of IGF2BP2 target transcripts in the brain, as this will be essential for evaluating both the therapeutic feasibility and potential side effects of targeting its RNA-binding domains. Only after a rigorous preclinical assessment of unwanted effects can IGF2BP2 itself be considered a viable direct drug target. In parallel, the measurement of IGF2BP2 expression in peripheral blood as a potential biomarker, or the non-invasive imaging of GLUT1 function, may offer alternative or complementary strategies for diagnosis and therapeutic monitoring in MDD.

## 4. Materials and Methods

### 4.1. Animals

All animals were raised and handled in accordance with the protocol approved by the institutional review board of the IBMS and Peking Union Medical College and were conducted according to the Beijing Administration Office of Laboratory Animals guidelines for the care and use of laboratory animals. Eight-week-old adult male C57BL/6J mice were purchased from Vital River Laboratory Animal Technology Co., Ltd. (Beijing, China). All the mice were maintained under standard housing conditions, with a 12 h dark/12 h light cycle, a temperature of 23 °C ± 2 °C, and free access to food and water. Mice were acclimatized for 1 week before the experiment.

### 4.2. CUMS Depression Mice Model Establishment

The chronic unpredictable mild stress (CUMS) model was established as previously described [61]. Briefly, mice were subjected to a 35-day paradigm during which they were exposed to 11 different mild stressors in a randomized and unpredictable manner, with 2 or 3 stressors administered per day during the light and/or dark phases. The 12 stressors applied were: elevated platform (1 m height, 30 min), restraint stress (in a tube with ventilation holes, 2–4 h), hot plate (45 °C, 10 min), daytime lights off (cages covered with black plastic for 6–8 h during the light phase), overnight illumination (continuous light overnight to disrupt circadian rhythm), cage tilt (45°, 6–8 h), crowding (15–20 mice per cage, 6–8 h), cold-water swimming (ice-water mixture, 3 min), food and water deprivation (up to 12 h), bedding deprivation (8–10 h) and wet bedding (12 h). Following the 35-day regimen, mice exhibit depression-like phenotypes including anhedonia (reduced sucrose preference) and behavioral despair (increased immobility in the forced swimming test), validating the successful establishment of the model.

### 4.3. Forced Swimming Test

Mice were individually placed in a 2 L beaker containing about 14 cm of water with a temperature of 23 °C to 25 °C, so the mice float without sinking. The total test duration was 6 min, and the immobility time within the last four minutes was collected for data analysis. To ensure objectivity, immobility time analysis was conducted by an experienced observer blinded to the experimental treatment. Only slight movement to keep balance or floating was considered as immobility.

### 4.4. Sucrose Preference Test

SPT was conducted to evaluate anhedonia-like behavior by measuring the innate preference for a 1% sucrose solution over plain water.

Housing conditions: Throughout the experiment, mice were group-housed (4–5 mice per cage) under standard conditions, except during the SPT procedure where temporary single housing was required. All mice were acclimated to single housing for 48 h prior to the test to minimize stress induced by isolation.

Procedure: Mice were individually housed and acclimated to two bottles containing 1% sucrose solution and water for 48 h, with the positions of the bottles switched after the first 24 h to avoid side preference. Following this adaptation period, mice underwent 24 h of water deprivation to ensure motivated drinking behavior. The test consisted of a 2 h exposure to two pre-weighed bottles: one filled with 1% sucrose solution and the other with water. The positions of the bottles were switched after the first hour. After the test, bottles were re-weighed, and the sucrose preference ratio was calculated as sucrose solution consumption divided by total liquid consumption.

Post-test housing: Immediately after the test, mice were returned to their original group-housing conditions to minimize prolonged isolation stress.

Rationale for behavioral testing order: In this study, SPT was conducted after FST to prevent single-housing stress from confounding other group-housed assessments. SPT requires individual housing for accurate measurement of fluid intake, and prolonged isolation is itself an anhedonia-inducing stressor, so SPT was placed at the end. Besides, SPT was separated from FST measurement by a ≥96 h interval (24 h rest + 48 h adaptation + 24 h water deprivation), far exceeding the typical recovery window for acute stress. This order is also consistent with previous studies [5,62].

### 4.5. rAAV Vector Construction and Packaging

All the rAAVs used in this study were constructed and packaged by Taitool Bioscience (Taitool Bioscience Co., Ltd., Shanghai, China). The titer of each rAAV preparatoin ranged from 3 × 10^12^ to 4 × 10^12^ vector genomes (v.g.)/mL. The final titer of rAAV microinjection was 1 × 10^12^ v.g./mL, and the injection volume was 1 microliter on each side of the hippocampus.

### 4.6. Hippocampal Stereotaxic Intracranial Injection

Eight-week-old male mice were stereotaxically injected with rAAVs into the hippocampus. The mice were anesthetized with 0.7% pentobarbital sodium intraperitoneally. One microliter of rAAV was microinjected bilaterally into the hippocampus (2.1 mm posterior to bregma, ±2.1 mm lateral to midline, and 1.7 mm ventral to dura) at a rate of 300 nanoliters/min with a Micro4 Syringe Pump Controller (World Precision Instruments, Sarasota, FL, USA). After 3 weeks, protein expression analysis and further experiments were performed.

### 4.7. Human Brain Samples

Tissues of the human brain were provided by the National Human Brain Bank, Chinese Academy of Medical Sciences and Peking Union Medical College, Beijing, China. Investigations were conducted in accordance with the principles according to the Declaration of Helsinki. Informed consent was provided by all the donors and their direct family members. The donation of human brain tissue was voluntary and free. It was also approved that the donated human brain tissue would only be used for scientific research. The autopsy and brain tissue collection was performed within 40 h after donor death.

The protocol was approved by the Ethics Committee of the Institute of Basic Medical Sciences Chinese Academy of Medical Sciences, School of Basic Medicine Peking Union Medical College. Approval Code: 2021109. Approval Date: 25 October 2021.

### 4.8. Transcriptome Microarray Analysis and RT-qPCR Validation

The microarray data analyzed in this study were generated as part of our previous research [5]. The dataset is publicly available in the NCBI Gene Expression Omnibus (GEO) under the accession number GSE201332.

Specifically, microarray profiling was performed on peripheral blood samples from an initial cohort of 20 MDD patients and 20 healthy controls. Total RNA was hybridized to Agilent 2100 human mRNA Microarrays. Differentially expressed genes were identified with |log_2_(fold change)| > 1 and adjusted *p* < 0.01. Among these, m6A reader genes were further examined.

Validation of IGF2BP2 upregulation in peripheral blood of MDD patients was furthered conducted on a second independent cohort with 31 MDD patients and 31 healthy controls by RT-qPCR.

### 4.9. RNA Stability

Two µg of IGF2BP2 overexpression plasmid or control vector was transfected into U87 cells cultured in 6-well plates when it reached 80–90% confluence. Then, 48 h later, the cells were treated with 5 μg/mL actinomycin D (A9415, Sigma-Aldrich, St. Louis, MO, USA) and harvested at 0, 3 and 6 h. Total RNA was then extracted using TRIzol (Thermo Fisher Scientific, Waltham, MA, USA), and 1 μg of total RNA was used for reverse transcription and qPCR.

### 4.10. RNA Immunoprecipitation (RIP) Assay, RNA Extraction and RT-qPCR

Briefly, U87 cells were cultured to 80–90% confluence in 10 cm dishes and then cross-linked with 1% formaldehyde for 10 min at room temperature to preserve protein-RNA interactions. After fixation, cells were lysed in complete RIP lysis buffer supplemented with protease inhibitor cocktail (B14001, Bimake, Shanghai, China) and RNase inhibitor (E00381, Thermo Fisher Scientific, Waltham, MA, USA).

The cell lysate was incubated with magnetic beads pre-conjugated with 5 μg of anti-IGF2BP2 antibody (ab124930, Abcam, Cambridge, UK) or normal rabbit IgG (#12-370, Millipore, Burlington, MA, USA) as a negative control, with gentle rotation overnight at 4 °C.

The following day, the bead-antibody complexes were washed six times with RIP wash buffer. The immunoprecipitated complexes were then treated with Proteinase K to digest the proteins and reverse the cross-links. Total RNA was isolated from the immunoprecipitates and Input control using TRIzol reagent according to the standard protocol.

The enriched RNA was then used to perform reverse transcription and qPCR by using the HiScript II One Step qRT-PCR SYBR Green Kit (Q221-01, Vazyme, Nanjing, China) on a LightCycler 96 system (Roche Diagnostics, Rotkreuz, Switzerland). The relative enrichment of Glut1 mRNA in the anti-IGF2BP2 group compared to the IgG control group was calculated using the 2^−ΔΔCt^ method after normalization to the Input sample.

### 4.11. Western Blot

Brain tissues or cell samples were lysed with RIPA lysis buffer and protease inhibitor cocktail (B14001, Bimake, Shanghai, China) in an order of homogenization, ultrasonication and centrifugation. After concentration analysis, proteins were mixed with loading buffer and denatured for 5 min at 95 °C. Next, 30 µg protein of each sample was further separated with 10% sodium dodecyl sulfate-polyacrylamide gel electrophoresis (SDS-PAGE) and then transferred onto nitrocellulose membranes. After antibody incubation, protein bands were finally visualized with enhanced chemiluminescence (ECL) reagent.

Primary antibodies were as follows: anti-IGF2BP2 (1:5000, ab124930, Abcam), anti-GLUT1 (1:50,000, ab115730, Abcam), anti-GAPDH (1:100,000, 60004-1-Ig, Proteintech, Rosemont, IL, USA).

Secondary antibodies were as follows: goat anti-rabbit IgG/HRP (SE134, Solarbio, Beijing, China), goat anti-mouse IgG/HRP (SE131, Solarbio, Beijing, China).

Sample selection for Western blot analyses was conducted as follows: For mice hippocampal Western blot experiments, three animals per group were randomly selected from the successfully established CUMS and control groups. Behavioral data from these subsets were consistent with the full-cohort results (i.e., significant differences between CUMS and control groups were preserved). The larger cohort sizes (n = 6–12 per group) for the establishment of CUMS model and behavioral tests were designed to accommodate the inherently greater inter-individual variability of stress tolerance and behaviors. This sampling strategy is consistent with common practice in rodent brain tissue Western blot.

### 4.12. Luciferase Assay

In the GLUT1 3′UTR fragment, there is only 1 m6A modification site with very high confidence, predicted by SRAMP. Wild type and mutant GLUT1 3′UTR were synthetized and inserted into pMIR-GLO vectors (Tsingke, Beijing, China). For dual-luciferase reporter assays, 50 ng pMIR-GLO vectors and 50 ng pcDNA3.1-IGF2BP2 or pcDNA3.1-mutant IGF2BP2 were co-transfected into U87 cells in 6-well plates. The mutant IGF2BP2-expressing vector contains the full-length IGF2BP2 coding region with mutations at K451E and K533E, which results in complete loss of m6A methylation recognition activity. Relative luciferase activities were assessed by Dual-Luciferase Reporter Assay (GM-040502A, GENOMEDITICH, Shanghai, China) after 48 h transfection.

**Glut1 3′UTR with wild-type m6A sites**

**(Highlighted “A” stands for predicted m6A site with high confidence**)

GGCAGCTGGATGAGACTTCCAAACCTGACAGATGTCAGCCGAGCCGGGCCTGGGGCTCCTTTCTCCAGCCAGCAATGATGTCCAGAAGAATATTCA**GGACT**TAACGGCTCCAGGATTTTAACAAAAGCAAGACTGTTGCTCAAATCTATTCAGACAAGCAACAGGTTTTATAATTTTTTTATTACTGATTTTGTTATTTTTATATCAGCCTGAGTCTCCTGTGCCCACATCCCAGGCTTCACCCTGAATGGTTCCATGCCTGAGGGTGGAGACTAAGCCCTGTCGAGACA

**Glut1 3′UTR with mutant m6A sites**

GGCAGCTGGATGAGACTTCCAAACCTGACAGATGTCAGCCGAGCCGGGCCTGGGGCTCCTTTCTCCAGCCAGCAATGATGTCCAGAAGAATATTCA**GGCCT**TAACGGCTCCAGGATTTTAACAAAAGCAAGACTGTTGCTCAAATCTATTCAGACAAGCAACAGGTTTTATAATTTTTTTATTACTGATTTTGTTATTTTTATATCAGCCTGAGTCTCCTGTGCCCACATCCCAGGCTTCACCCTGAATGGTTCCATGCCTGAGGGTGGAGACTAAGCCCTGTCGAGACA

### 4.13. Statistical Analysis

Statistical analyses were performed using Prism 8.0, with an alpha level of *p* < 0.05 considered statistically significant. Data are presented as mean ± standard error of the mean (SEM). For comparisons between two groups, Welch’s *t*-test was used (Figure 1B–I, Figure 2D, Figure 3A,B,D,E and Figure 4A). For comparisons involving more than two groups with a single independent variable, one-way ANOVA followed by Bonferron’s post hoc test was applied (Figure 2F,G, Figure 3G, Figure 4C,E, Figure 5F,G and Figure S1A,B). For experiments involving two independent variables, two-way ANOVA with Bonferroni’s multiple comparisons test was performed (Figure 3C,F, Figure 4B, and Figure 5D). Standard deviations were assessed using the F test for two-group comparisons and the Brown–Forsythe and Welch ANOVA tests for multiple-group comparisons. All data were analyzed using parametric tests after confirming an approximately normal distribution. For all tests herein, * *p* < 0.05, ** *p* < 0.01, *** *p* < 0.001, **** *p* < 0.0001. N numbers are reported in the corresponding figure legends.

## 5. Conclusions

In conclusion, our study identifies the m6A reader IGF2BP2 as an epigenetic regulator of depressive-like behaviors, revealing a mechanism by which IGF2BP2 post-transcriptionally represses astrocytic GLUT1 in an m6A-dependent manner, thereby impairing hippocampal glucose uptake. Hippocampal knockdown of Igf2bp2 produces antidepressant-like effects, and overexpression of Glut1 may rescue both the behavioral and metabolic deficits. These results converge to establish the IGF2BP2-GLUT1 axis as a previously unrecognized pathway at the interface of epitranscriptomic regulation and brain energy metabolism in depression. These findings not only deepen our understanding of the epigenetic and metabolic underpinnings of depression but also highlight promising targets for future therapeutic intervention.

## Data Availability

The microarray dataset analyzed in this work is now openly available in the NCBI Gene Expression Omnibus (GEO) under accession number GSE201332 (https://www.ncbi.nlm.nih.gov/geo/query/acc.cgi?acc=GSE201332, accessed on 25 October 2026). The raw data supporting the conclusions of this article will be made available by the authors on request.

## Supplementary Materials

The following supporting information can be downloaded at: https://www.mdpi.com/article/10.3390/ijms27219803/s1.

## Abbreviations

The following abbreviations are used in this manuscript:

MDD	Major depressive disorder
CUMS	Chronic unpredictable mild stress
IGF2BP2	Three letter acronym
GLUT1	Glucose transporter 1
m6A	N6-methyladenosine
YTH	YT521-B homology
RRM	RNA recognition motif
KH	K homology
FST	Forced swimming test
SPT	Sucrose preference test
PFC	Prefrontal cortex
HIP	Hippocampus
AMY	Amygdala
rAAV	Recombinant adeno-associated virus
RIP	RNA immunoprecipitation
